# Intermuscular and intramuscular distributions of sprint-induced thigh muscle activation in male sprinters: a T2-weighted MRI study

**DOI:** 10.3389/fphys.2026.1904100

**Published:** 2026-09-16

**Authors:** Haruto Arai, Tadashi Suga, Masafumi Terada, Yuki Kusagawa, Keishi Kuroki, Masahiro Umeda, Tadao Isaka

**Affiliations:** 1 Graduate School of Sports and Health Science, Ritsumeikan University, Kusatsu, Japan; 2 Faculty of Sport and Health Science, Ritsumeikan University, Kusatsu, Japan; 3 Institute of Advanced Research for Sport and Health Science, Ritsumeikan University, Kusatsu, Japan; 4 Research Organization of Science and Technology, Ritsumeikan University, Kusatsu, Japan; 5 Meiji University of Integrative Medicine, Nantan, Japan

**Keywords:** adductor magnus, functional magnetic resonance imaging, hamstring strain injury, semitendinosus, sprint performance

## Abstract

Superior sprint performance is associated with greater activation of the lower-limb muscles, particularly the thigh muscles, during sprinting. T2-weighted magnetic resonance imaging (MRI) is an ideal method for assessing spatial patterns of thigh muscle activation in response to dynamic whole-body movements. In this T2-weighted MRI study, we examined the intermuscular and intramuscular distributions of sprint-induced activation across 13 thigh muscles in male sprinters using a low-volume sprint protocol designed to preserve maximal performance while minimizing fatigue. Specifically, we evaluated intermuscular activation patterns and intramuscular regional differences in thigh muscle activation. Thirty-two well-trained male collegiate sprinters (100-m seasonal best time: 11.02 ± 0.28 s) completed the low-volume sprint protocol consisting of three maximal 60-m sprints with 2-min recovery intervals on an indoor track. T2-weighted MRI scans were acquired before and after the sprint protocol. Intermuscular activation patterns were assessed by comparing the percentage change in T2 across 13 thigh muscles. Intramuscular regional differences were assessed by comparing T2 changes among regions within each muscle. During the low-volume sprint protocol, 60-m sprint times did not differ significantly among the three maximal sprints (*P* = 0.349). Despite this minimized approach, significant T2 increase were observed across 12 of the 13 thigh muscles (3.1–19.1%, all *P* < 0.05), with the greatest increase in the semitendinosus. In particular, T2 changes in the hamstrings and adductors were markedly greater than those in the quadriceps femoris. Furthermore, significant regional differences in T2 changes were identified in six of the 11 muscles analyzed (all *P* < 0.05). Notably, the T2 change in the proximal region of the biceps femoris long head was significantly greater than those in the middle and distal regions (both *P* < 0.05), which may be relevant to the regional susceptibility to hamstring strain injury. These findings suggest that maximal sprint performance in male sprinters involves complex muscle recruitment mechanisms characterized by intermuscular and intramuscular distributions of thigh muscle activation. A better understanding of these features may contribute to the development of novel sprint strategies to enhance sprint performance and prevent sprint-related sports injuries in sprinters.

## Introduction

Superior sprint performance requires the ability to generate and regulate large lower-limb forces during maximal sprinting (Dorn et al., 2012; Schache et al., 2013; Slawinski et al., 2017; Healy et al., 2022). In particular, the thigh muscles play pivotal roles in generating propulsive forces and regulating decelerative forces throughout the sprint cycle (Dorn et al., 2012; Schache et al., 2012; Higashihara et al., 2018). Indeed, morphological characteristics of several thigh muscles in sprinters are associated with sprint performance (Ema et al., 2018; Miller et al., 2021; Takahashi et al., 2021; Kawama et al., 2024), possibly because these muscles contribute to greater force production during sprinting (Morin et al., 2015; Ema et al., 2018; Nagahara and Murata, 2024). Consistent with these findings, previous studies have extensively examined thigh muscle activity during sprinting using surface electromyography (Howard et al., 2018). However, surface electromyography has methodological limitations in simultaneously measuring muscle activity across all thigh muscles, particularly small and/or deep muscles (Farina et al., 2025), although these muscles may play important roles in sprinting (Miller et al., 2021). Furthermore, high-speed dynamic whole-body movements such as sprinting may increase the likelihood of signal contamination caused by intermuscular crosstalk, raising concerns about the validity of measurements of individual thigh muscle activity. Consequently, previous studies examining muscle activity during sprinting have largely been limited to thigh muscles that are both relatively large and superficially located (Howard et al., 2018). These methodological limitations of surface electromyography pose substantial challenges to assessing thigh muscle activity during maximal sprinting.

T2-weighted magnetic resonance imaging (MRI) is a useful noninvasive tool for assessing muscle activation based on the increase in transverse relaxation time (i.e., T2) following exercise (Adams et al., 1992). It is well established that exercise-induced changes in muscle T2 measured using T2-MRI are associated with muscle activity assessed using surface electromyography (Adams et al., 1992; Kinugasa and Akima, 2005) and with changes in muscle energy metabolism (e.g., phosphocreatine depletion and inorganic phosphate accumulation) assessed using phosphorus magnetic resonance spectroscopy (Weidman et al., 1991; Cheng et al., 1995). Previous studies using T2-weighted MRI have reported spatial patterns of thigh muscle activation in response to various dynamic lower-limb exercises (Dooley et al., 2020). However, research examining thigh muscle activation during sprinting remains limited because a specialized research environment is required to allow T2-weighted MRI measurements immediately after exercise. To the best of our knowledge, Bourne et al. (2021) were the first to report activation in the hamstrings after a sprint protocol in male athletes, although their participants were not sprinters. Subsequently, Yoshimoto et al. (2022) examined activation in the 13 thigh muscles after a sprint protocol in male sprinters and demonstrated non-uniform activation patterns across the thigh muscles, with the semitendinosus showing the greatest activation, consistent with findings from morphological studies (Handsfield et al., 2017; Miller et al., 2021; Takahashi et al., 2021, 2022; Kawama et al., 2024). Importantly, the sprint protocols used in these two previous studies may have involved a relatively high-volume. Bourne et al. (2021) employed a sprint protocol consisting of a total of 18 repetitions of 40-m sprints with 30-s recovery intervals. Yoshimoto et al. (2022) used a sprint protocol consisting of a total of 9 repetitions of 60-m sprints with 30-s recovery intervals. These sprint protocols may induce physical and muscular fatigue, as indicated by decreases in sprint times among repetitions (Bourne et al., 2021; Yoshimoto et al., 2022), which may impair sprint mechanics (Edouard et al., 2018). In addition, previous studies have demonstrated that whole-body and local exercises inducing marked muscle fatigue may alter the activation patterns among the thigh muscles, particularly within muscle groups and between synergistic muscles, as assessed by T2-weighted MRI (Akima et al., 2002; Endo et al., 2007). Considering these findings, high-volume sprint protocols may be useful for detecting distinct activation patterns among the thigh muscles (Bourne et al., 2021; Yoshimoto et al., 2022), but the resulting muscle fatigue may alter these activation patterns (Edouard et al., 2018). Therefore, the development of a low-volume sprint protocol that does not impair sprint performance and minimizes muscle fatigue may provide a valid method for examining thigh muscle activation during sprinting in athletes, particularly sprinters.

Previous studies using T2-weighted MRI have reported that exercise-induced thigh muscle activation exhibits non-uniform patterns not only across muscles but also across regions within each muscle (Akima et al., 2004; Mendez-Villanueva et al., 2016; Mechó et al., 2024). For example, Akima et al. (2004) reported greater activation in the distal region than in the proximal region of the rectus femoris after local knee extension exercise. Such intramuscular regional differences in thigh muscle activation are consistent with previous findings of region-specific muscle hypertrophy following long-term training (Ema et al., 2013), suggesting a potential relationship between acute muscle activation and chronic muscle hypertrophy not only among muscles but also within individual muscles (Wakahara et al., 2012, 2013, 2017). Despite these findings, intramuscular regional distributions of thigh muscle activation following high-speed dynamic whole-body movements such as sprinting remain unknown. Using surface electromyography, Cerone et al. (2023) determined greater activity in the proximal region than in other regions of the biceps femoris during the late swing phase of an 80-m submaximal sprint. Additionally, a morphological study by Takahashi et al. (2022) demonstrated region-specific hypertrophy in several hamstring and adductor muscles in sprinters compared with untrained individuals, with particularly greater hypertrophy in the proximal and middle regions than in the distal region of the semitendinosus. Therefore, these findings suggest that non-uniform intramuscular regional distributions of thigh muscle activation may occur during sprinting, which could further advance our understanding of sprinting mechanics.

To address these issues, in this T2-weighted MRI study, we examined the intermuscular and intramuscular distributions of sprint-induced activation across 13 thigh muscles in male sprinters using an original low-volume sprint protocol. Specifically, we evaluated intermuscular activation patterns and intramuscular regional differences of thigh muscle activation. We expected that our sprint protocol, consisting of three 60-m sprints separated by 2-min recovery intervals and representing a relatively low sprint volume, would not impair maximal sprint performance and would elicit sufficient pre- to post-exercise T2 changes to detect clear differences in muscle activity among the thigh muscles. Furthermore, with this minimal yet appropriate approach, we expected that distinct intermuscular and intramuscular distributions of thigh muscle activation would be identified, thereby facilitating the interpretation of previous findings and providing new insights that may contribute to the development of novel sprint strategies for sprinters.

## Methods

### Participants

Before this study, an *a priori* sample size calculation was conducted for the planned one-factor repeated-measures design with adjustment for multiple comparisons for the primary outcome measure (i.e., differences in muscle activity among the 13 thigh muscles). Assuming a medium effect size of 0.25 (Cohen’s *f*), a significance level of 0.05 (α = 0.05), and a statistical power of 80% (*β* = 0.20), the required sample size was estimated to be at least 24 participants. Accordingly, the present study recruited 32 well-trained male collegiate sprinters (age: 19.9 ± 1.6 years, body height: 173.8 ± 5.0 cm, body mass: 66.3 ± 4.8 kg) to account for potential dropouts and possible exclusions due to unusable or extreme data. Their 100-m personal best and seasonal best times were 10.93 ± 0.27 s and 11.02 ± 0.28 s, respectively. All participants had at least 4 years of competitive experience (8.4 ± 2.8 years) and engaged in sprint-specific training for ≥ 3 hours per day, 5 days per week. The participants were free from known cardiovascular, metabolic, neurological, and orthopedic disorders and reported no history of musculoskeletal injury within the previous 3 months. None of the participants had contraindications to MRI (e.g., ferromagnetic implants, pacemakers, or severe claustrophobia). To minimize the residual effects of prior physical and muscular fatigue, participants were instructed to refrain from their regular sprint-specific training and other high-intensity exercises for 36 hours before the experimental session. This study was approved by the Ethics Committee of Ritsumeikan University (BKC-LSMH-2023-025) and conducted in accordance with the Declaration of Helsinki. All participants were fully informed of the purpose and potential risks of the study and provided written informed consent prior to participation.

### Experimental procedure

On the experimental day, after arriving at the MRI laboratory, participants first lay supine in the MRI scanner for 20 min to ensure resting conditions, followed by pre-exercise T2-weighted MRI scan. Thereafter, participants performed standard and sprint-specific warm-up sessions totaling approximately 50 min, which were designed to prepare them for maximal sprinting while minimizing excessive physical and muscular fatigue. The standard warm-up consisted of 5 min of low-intensity ergometer cycling, dynamic stretching (e.g., hip, knee, and ankle mobilization), dynamic stretching with forward progression (e.g., walking lunges and leg swings), and three types of jump tasks (e.g., squat jumps, countermovement jumps, and rebound jumps), each performed twice, with a total duration of 30 min. The sprint-specific warm-up consisted of three 30-m sprints: one performed at approximately 50% of maximal effort while wearing running shoes, followed by one performed at approximately 50% and one performed at approximately 70% of maximal effort while wearing each participant’s own spike shoes. The low-volume sprint protocol was then conducted 10 min after completion of the warm-up sessions. Immediately after the final sprint trial, participants sat or lay on a cart and were transported from the indoor track to the MRI laboratory to avoid metabolic loads associated with walking and other lower-limb movements. The time interval between completion of the final sprint trial and the start of the post-exercise MRI scan was exactly 5 min for all participants.

### Low-volume sprint protocol

The sprinting distance used in the low-volume sprint protocol was set at 60 m on an indoor straight synthetic track, in accordance with the methods of a previous study (Yoshimoto et al., 2022) and evidence that general sprinters reach maximal running velocity between 50 and 60 m (Slawinski et al., 2017). The protocol consisted of three maximal 60-m sprints separated by 2-min recovery intervals, with the number of sprints and recovery duration determined in consideration of the results of a series of preliminary experiments. All sprint trials were initiated from a crouched three-point start position. Participants wore their own sprint spike shoes, which they regularly used during training and competition. Sprint times during the three trials were measured using a wireless photocell timing system (Witty, Microgate, Bolzano, Italy), with photocells positioned at 0, 30, and 60 m to record passing times at each point. Sprint time variables included the 0–30 m and 30–60 m interval times, representing the acceleration and maximal velocity phases, respectively, as well as the total 60-m sprint time. Participants were instructed to perform each sprint trial with maximal effort and to maintain maximal speed through the 60-m mark. The coefficients of variation across the three trials were 0.8 ± 0.7% for the 60-m sprint time, 1.0 ± 0.6% for the 0–30-m sprint time, and 0.7 ± 0.5% for the 30–60-m sprint time, respectively, indicating very low trial-to-trial variability and suggesting that sprint performance was consistently maintained across the three trials.

### T2-weighted MRI

T2-weighted images of the right thigh were acquired using a 3.0-T whole-body MRI system (MAGNETOM Skyra; Siemens Healthineers, Erlangen, Germany). Participants were positioned supine with the hips and ankles in neutral positions and the knees fully extended. They were instructed to relax and to avoid voluntary contractions or unnecessary movement of the right leg during imaging. A multi-spin-echo sequence was used to obtain T2-weighted images with the following parameters: 15 axial slices, a slice thickness of 5.0 mm, an inter-slice gap of 15.0 mm, a field of view of 256 × 256 mm, a repetition time of 2500 ms, and echo times of 10, 20, 30, 40, 50, and 60 ms. The scan time for T2 acquisition was 5 min 10 s. The imaging range was defined along the longitudinal axis of the femur. The distance from the greater trochanter to the lateral femoral epicondyle was measured in each participant (39.0 ± 2.0 cm), and the midpoint (50%) of this segment was identified. Axial images were then acquired over a 30-cm region centered at this midpoint (± 15 cm), covering the proximal and distal portions of all thigh muscles analyzed. The following 13 thigh muscles were analyzed: rectus femoris, vastus lateralis, vastus medialis, vastus intermedius, sartorius, adductor brevis, adductor longus, adductor magnus, gracilis, semimembranosus, semitendinosus, long head of the biceps femoris, and short head of the biceps femoris.

All image analyses were performed using ImageJ (Version 1.54; National Institutes of Health, MD, USA). MRI data were exported as DICOM files, and pixel-wise T2 values were calculated by fitting the signal intensity-echo time relationship to a mono-exponential decay model. For each muscle, a region of interest (ROI) was manually delineated to encompass the entire muscle cross-section in each slice. Particular care was taken to exclude non-contractile tissues (e.g., subcutaneous fat and fascia), visible blood vessels, and areas affected by motion or other artefacts from the ROI. Based on each participant’s thigh length (i.e., the distance from the greater trochanter to the lateral femoral epicondyle), muscle-specific T2 values were first obtained at three relative longitudinal positions corresponding to 30%, 50%, and 70% of the thigh length (**Figure 1**). The representative T2 value for each muscle was then defined as the value obtained from the axial slice that most closely corresponded to the anatomical muscle belly, following the approach described previously (Yoshimoto et al., 2022). However, the sartorius was analyzed using the distal region because elevated T2 values attributable to artefacts were already observed in the proximal and middle regions at the pre-exercise time point, and it was therefore excluded from the intramuscular distribution analysis described below. The change in T2 for each muscle from pre- to post-exercise in the low-volume sprint protocol was calculated as follows: T2 change (%) = (post − pre)/pre × 100. These values were used to assess the intermuscular distributions of thigh muscle activation after maximal sprinting. In addition, intramuscular distributions of muscle activation were assessed by examining region-specific differences in T2 change within each of the 11 thigh muscles, excluding the sartorius and adductor brevis; the latter was excluded because it does not span both the middle and distal regions. The intramuscular regional differences in eight muscles, excluding the semimembranosus, short head of the biceps femoris, and adductor longus, were examined across three regions (i.e., proximal, middle, and distal regions). The semimembranosus and the short head of the biceps femoris were analyzed only in the middle and distal regions because the proximal ROIs could not be delineated to encompass the entire muscle cross-section in some participants. Similarly, the adductor longus was analyzed only in the proximal and middle regions because the distal ROI could not be delineated in some participants.

**Figure 1:**
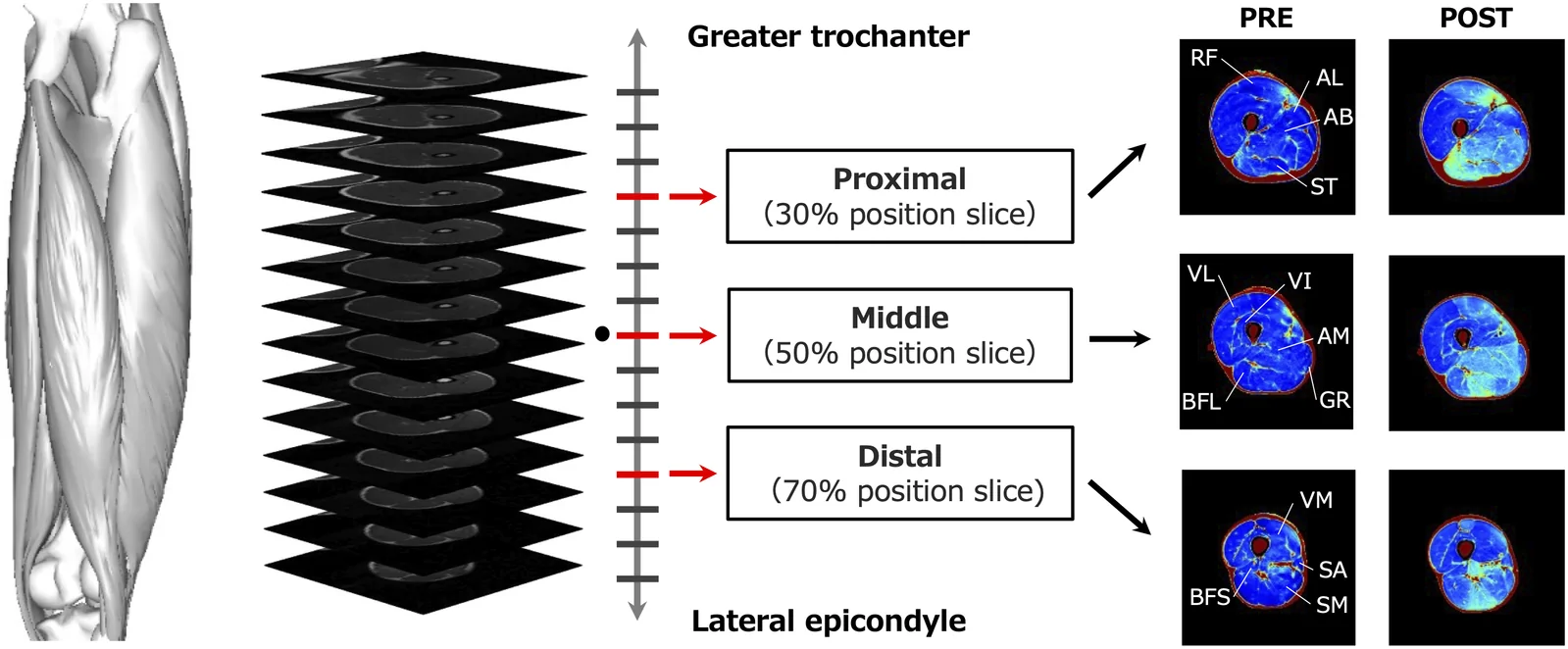
Analytical workflow for T2-weighted magnetic resonance imaging of thigh muscle activation. The T2 values were analyzed at three representative slice locations along the thigh length, defined as the distance between the greater trochanter and the lateral epicondyle. These slice locations corresponded to 30% (proximal), 50% (middle), and 70% (distal) of the thigh length. Representative T2 maps at each slice level before (PRE) and after (POST) the low-volume sprint protocol are shown. The slice levels used in the primary analysis across all 13 thigh muscles were as follows: the proximal 30% for the rectus femoris (RF), adductor brevis (AB), adductor longus (AL), and semitendinosus (ST); the middle 50% for the vastus lateralis (VL), vastus intermedius (VI), adductor magnus (AM), gracilis (GR), and biceps femoris long head (BFL); and the distal 70% for the vastus medialis (VM), sartorius (SA), semimembranosus (SM), and biceps femoris short head (BFS).

All muscle ROIs were manually traced by a single experienced analyst who was not blinded to the pre- and post-exercise conditions. To assess the intra-rater reliability of manual ROI tracing, the analyst performed the measurements twice on pre-exercise images from 12 randomly selected participants in a preliminary analysis. The coefficients of variation ranged from 0.0 ± 0.0% to 0.6 ± 0.8%, and the ICC(3,1) values ranged from 0.831 to 1.000, indicating good to excellent reliability according to the criteria of Koo and Li (2016).

### Statistical analysis

Data are presented as means and standard deviations. All statistical analyses were performed using RStudio (version 2023.12.0 + 369; Posit Software PBC, Boston, MA, USA). Statistical significance was set at *P* < 0.05. A one-way repeated-measures analysis of variance was used to examine differences in sprint time variables among the three 60-m sprints performed during the low-volume sprint protocol. To account for within-subject variability due to repeated measurements, a two-factor repeated-measures linear mixed-effects model fitted using restricted maximum likelihood was used to determine the main effects of time and muscle, as well as the time × muscle interaction, on pre- and post-exercise T2 values. Random intercepts were included for participants and for participant-by-muscle combinations. When a significant interaction was detected, *post hoc* simple-effects analyses were performed using estimated marginal means with Bonferroni adjustment to determine pre- to post-exercise T2 changes within each muscle. Moreover, intermuscular differences in T2 changes were assessed using one-factor repeated-measures linear mixed-effects models fitted using restricted maximum likelihood, with muscle as a fixed effect, participant as a random intercept, and pre-exercise whole-muscle T2 values included as a covariate to account for baseline differences among muscles. When a significant main effect was observed, *post hoc* pairwise comparisons were performed with Tukey adjustment for multiple comparisons. Furthermore, intramuscular differences in T2 changes were assessed separately for each muscle using one-factor repeated-measures linear mixed-effects models fitted using restricted maximum likelihood, with region as a fixed effect, participant as a random intercept, and region-specific pre-exercise T2 values included as a covariate to account for baseline differences among regions. The same modeling approach was applied to muscles with only two regions, in which the fixed effect had two levels. When a significant main effect was detected, *post hoc* pairwise comparisons among three regions were performed using Tukey adjustment for multiple comparisons, whereas a single planned contrast was used for comparisons between two regions.

## Results

### Low-volume sprint protocol

All participants completed a low-volume sprint protocol. A one-way repeated-measures analysis of variance revealed no significant differences in 60-m sprint time among the three maximal sprint trials (*P* = 0.349; **Figure 2**). No significant differences were also observed for sprint times over 0–30 m and 30–60 m (*P* = 0.608 and *P* = 0.250, respectively).

**Figure 2:**
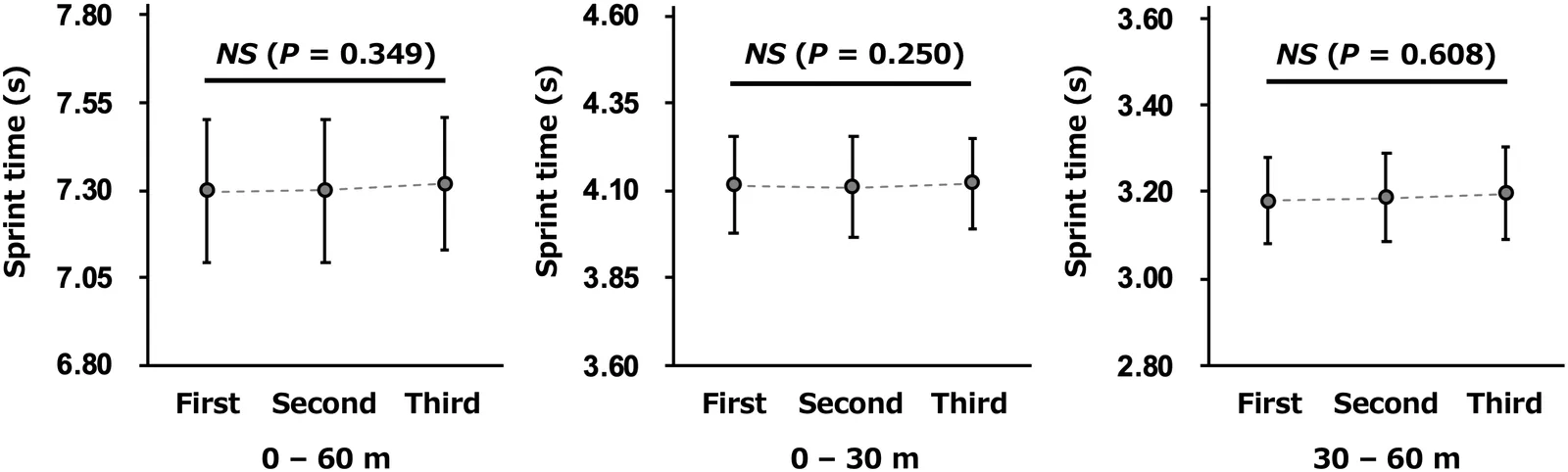
Changes in sprint time variables across three trials during the low-volume sprint protocol. Data are represented as means and standard deviations. No significant differences (*NS*) were observed for the three sprint time variables.

### Intermuscular distribution of thigh muscle activation

A two-factor repeated-measures linear mixed-effects model revealed a significant main effect of time on the pre- to post-exercise change in T2 following the low-volume sprint protocol (*P* < 0.001). The main effect of muscle was not significant (*P* = 0.452), and a significant time × muscle interaction was detected (*P* < 0.001). *Post hoc* simple-effects analyses with Bonferroni adjustment showed significant T2 increases in 12 of the 13 muscles (adjusted *P* < 0.05; **Table 1**), except for the vastus medialis (adjusted *P* = 0.238). In addition, a one-factor repeated-measures linear mixed-effects model revealed a significant main effect of muscle on the T2 change (*P* < 0.001). The semitendinosus exhibited the greatest T2 change (19.1%), followed by the adductor magnus (14.4%). *Post hoc* pairwise comparisons with Tukey adjustment for multiple comparisons showed that the T2 change in the semitendinosus was significantly greater than that in all other muscles (all *P* < 0.05; **Figure 3**), except for the adductor magnus (*P* = 0.911). The T2 change in the adductor magnus was significantly greater than that in the sartorius, adductor brevis, gracilis, semimembranosus, biceps femoris short head, and all four quadriceps femoris muscles (all *P* < 0.05). Overall, the T2 changes in most hamstring and adductor muscles were markedly greater than those in the quadriceps femoris muscles.

**Table 1:** T2 values for the 13 thigh muscles before (PRE) and after (POST) the low-volume sprint protocol.

Muscle	PRE		POST		adjusted *P*
	Mean (SD)	95% CI	Mean (SD)	95% CI	
RF	36.5 (1.4)	36.0−37.0	39.1 (2.1)	38.4−39.9	< 0.001
VL	38.0 (1.1)	37.6−38.4	39.3 (1.1)	38.8−39.7	0.001
VM	38.7 (1.2)	38.3−39.1	38.9 (1.5)	38.9−40.0	0.238
VI	37.9 (0.8)	37.6−38.2	38.6 (1.4)	38.6−39.6	0.002
SA	38.7 (2.7)	37.7−39.7	41.1 (2.6)	41.1−43.0	< 0.001
AB	36.7 (0.9)	36.4−37.0	40.2 (2.8)	40.2−42.2	< 0.001
AL	37.4 (1.3)	37.0−37.9	41.0 (2.6)	41.0−42.9	< 0.001
AM	38.4 (1.2)	38.0−38.8	43.1 (2.2)	43.1−44.7	< 0.001
GR	35.9 (1.0)	35.6−36.3	40.0 (2.4)	40.0−41.8	< 0.001
SM	38.3 (1.4)	37.8−38.8	41.1 (1.9)	40.4−41.8	< 0.001
ST	36.9 (1.2)	36.5−37.3	43.9 (4.3)	42.4−45.5	< 0.001
BFL	38.0 (1.4)	37.5−38.5	42.8 (2.4)	41.9–43.6	< 0.001
BFS	37.4 (1.5)	36.8−37.9	41.4 (2.1)	40.5−41.9	< 0.001

Values are presented as means and standard deviations (SD). Significant pre- to post-exercise changes in T2 after Bonferroni adjustment are indicated (adjusted *P* < 0.05). CI, confidence interval; RF, rectus femoris; VL, vastus lateralis; VM, vastus medialis; VI, vastus intermedius; SA, sartorius; AB, adductor brevis; AL, adductor longus; AM, adductor magnus; GR, gracilis; SM, semimembranosus; ST, semitendinosus; BFL, biceps femoris long head; BFS, biceps femoris short head.

**Figure 3:**
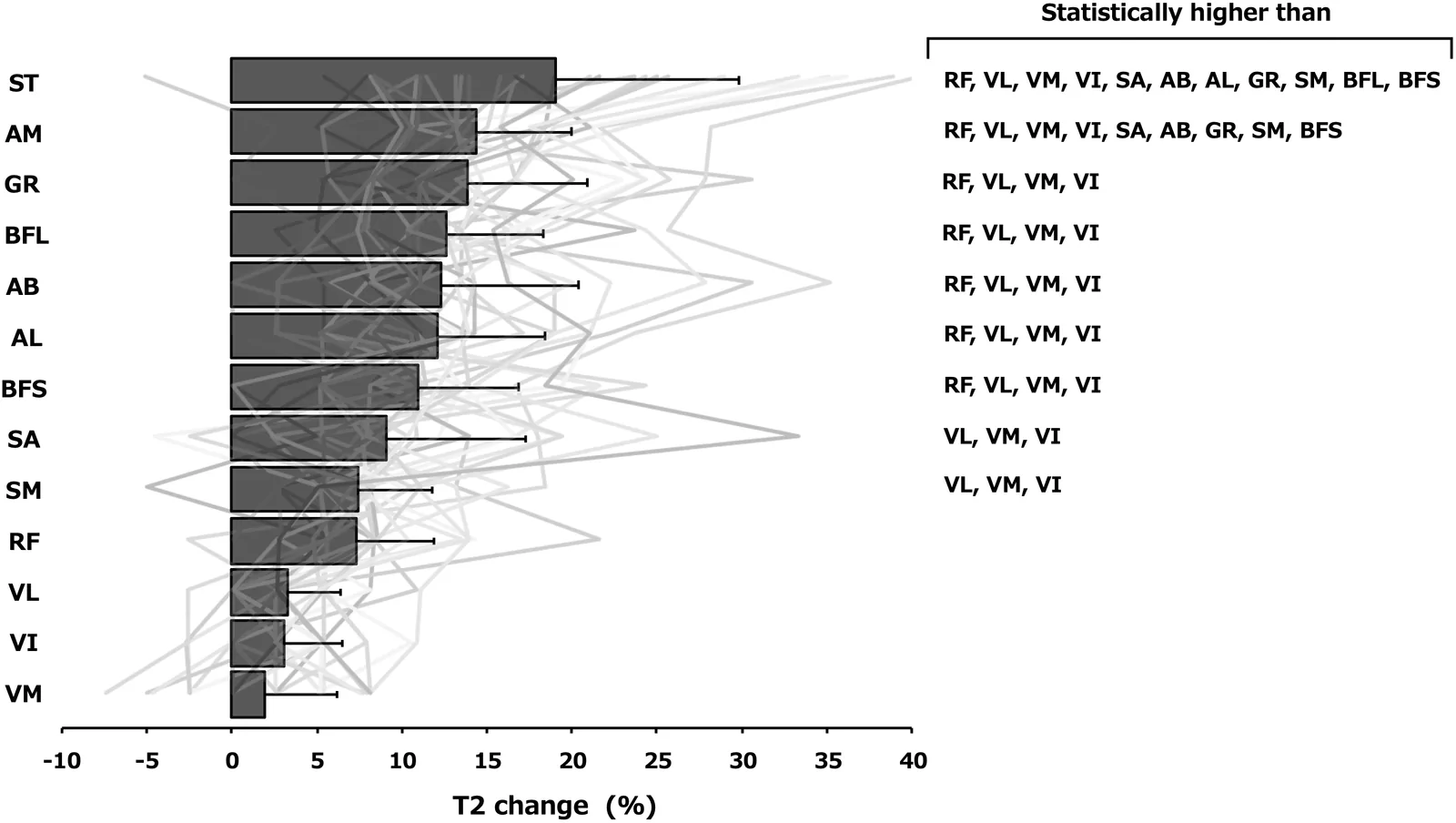
Comparison of muscle activation among the 13 thigh muscles and participant-specific intermuscular plots. Black bars represent group means and standard deviations. Gray lines indicate individual participant plots. Statistical significance for multiple comparisons was set at an adjusted *P* < 0.05.

### Intramuscular distributions of thigh muscle activation

A repeated-measures linear mixed-effects model revealed a significant main effect of region in the rectus femoris, vastus lateralis, adductor magnus, semimembranosus, semitendinosus, and biceps femoris long head (all *P* < 0.05). In *post hoc* pairwise comparisons with Tukey adjustment for multiple comparisons, the rectus femoris showed a significantly greater T2 change in the distal region than in the proximal region (*P* = 0.022; **Figure 4**). The vastus lateralis showed significantly greater T2 changes in the middle and distal regions than in the proximal region (both *P* < 0.001), and the distal region was also significantly greater than the middle region (*P* < 0.001). The adductor magnus showed significantly greater T2 changes in the middle and distal regions than in the proximal region (both *P* ≤ 0.001), and the distal region was also significantly greater than the middle region (*P* < 0.001). In the semimembranosus, which was compared between two regions, the T2 change was significantly greater in the middle region than in the distal region (*P* < 0.001). The semitendinosus showed significantly greater T2 changes in the middle and distal regions than in the proximal region (both *P* < 0.001), and the middle region was also significantly greater than the distal region (*P* = 0.043). The biceps femoris long head showed a significantly greater T2 change in the proximal region than in the middle and distal regions (both *P* < 0.001), and the middle region was also significantly greater than the distal region (*P* = 0.002).

**Figure 4:**
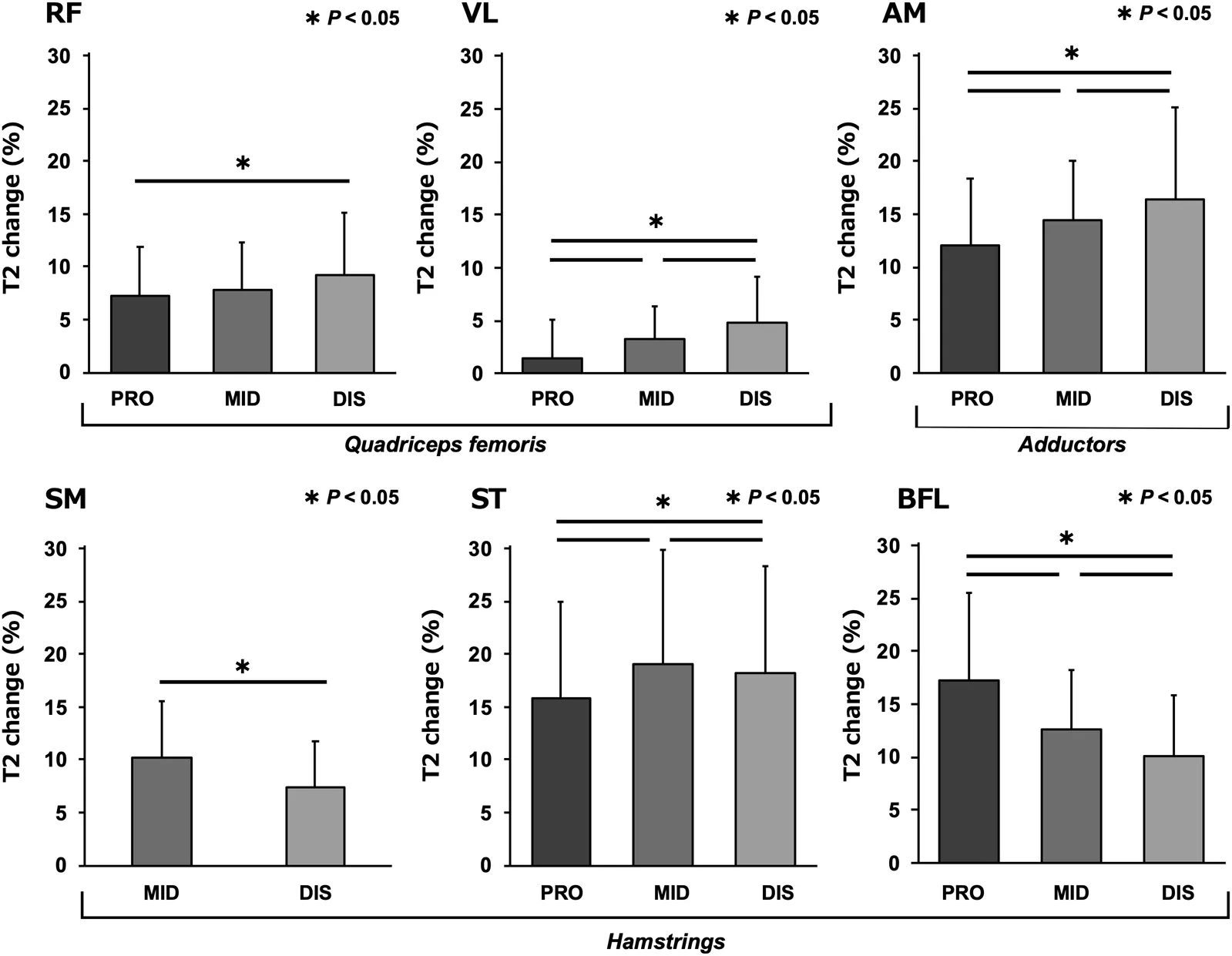
Regional differences in muscle activation within selected thigh muscles. Data are represented as means and standard deviations. This figure shows the results for six of the 11 analyzed thigh muscles, excluding the SA and AB, in which significant intramuscular regional differences were observed. The five muscles, excluding the SM, were compared among three regions: proximal (PRO), middle (MID), and distal (DIS). In contrast, the SM was compared only between the MID and DIS regions because the proximal region of interest could not be delineated to encompass the entire muscle cross-section in some participants. Asterisks indicate a significant difference at an adjusted *P* < 0.05.

## Discussion

This study examined thigh muscle activation, as assessed by T2-weighted MRI, in response to maximal sprinting using a novel low-volume sprint protocol in male sprinters. As expected, the sprint protocol did not significantly worsen sprint times across the three 60-m sprint trials, suggesting that participants were able to maintain sprint performance across the three sprints. Despite this minimized approach, we observed significant pre- to post-exercise increases in T2 in 12 of the 13 thigh muscles, with the greatest increase in the semitendinosus. Furthermore, we identified significant regional differences in T2 changes across six muscles, indicating that neuromuscular activity and metabolic loading during maximal sprinting are non-uniform both across and within the thigh muscles. Therefore, these findings suggest that, in line with our expectations, maximal sprint performance in male sprinters is achieved through complex muscle recruitment mechanisms characterized by non-uniform intermuscular and intramuscular distributions of thigh muscle activation, which can be detected using a low-volume sprint protocol while maintaining sprint performance.

The present study used an original sprint protocol consisting of three 60-m maximal sprints with 2-min recovery intervals, which was lower in volume than the sprint protocols used in previous studies (Bourne et al., 2021; Yoshimoto et al., 2022). The percentage increase in T2 following the low-volume sprint protocol ranged from 3.1% to 19.1% across the 12 thigh muscles that showed significant changes, with the greatest increase observed in the semitendinosus, followed by the adductor magnus (14.4%). Therefore, the T2 changes observed in the present study were generally lower than those reported in the previous study (Yoshimoto et al., 2022), possibly because muscle fatigue may have been less pronounced; nevertheless, the present findings were partially consistent with the previous results despite the use of a low-volume protocol. Notably, the present findings support the notion that the semitendinosus exhibits the greatest sprint-induced muscle activation among the thigh muscles, which is consistent with previous reports using T2-weighted MRI as well as surface electromyography (Higashihara et al., 2018; Yoshimoto et al., 2022). The semitendinosus is a biarticular muscle that acts as a hip extensor and knee flexor and likely contributes to the generation of greater hip extension and knee flexion torques during sprinting, which are key kinetic variables for superior sprint performance (Dorn et al., 2012; Schache et al., 2012; Morin et al., 2015; Higashihara et al., 2018; Nagahara and Murata, 2024). Additionally, compared with other thigh muscles, the semitendinosus shows greater hypertrophy in sprinters than in untrained individuals (Handsfield et al., 2017; Miller et al., 2021; Takahashi et al., 2022), potentially reflecting greater neuromuscular activity and metabolic loading during sprinting. Furthermore, the association between semitendinosus size and sprint performance in male sprinters has been well documented (Miller et al., 2021; Takahashi et al., 2021; Kawama et al., 2024). Taken together, the present findings suggest that semitendinosus activation during sprinting may play a key role in achieving superior sprint performance in male sprinters.

In the present study, the T2 change in the adductor magnus was the second largest among the 13 thigh muscles and did not differ significantly from that in the semitendinosus. Yoshimoto et al. (2022) reported a significantly greater T2 change in the semitendinosus than in the adductor magnus during sprinting in male sprinters. Thus, the present findings partially differ from those reported in the previous study, possibly because of fatigue-related differences in muscle recruitment patterns resulting from the different numbers of sprint repetitions. With an increasing number of sprints, the contribution of the primary hip extensors (e.g., the gluteus maximus) may have decreased, thereby increasing the relative demand on the semitendinosus (Matsunaga et al., 2021) and potentially resulting in a greater T2 increase in the semitendinosus than in the adductor magnus. In contrast, although the adductor magnus is also considered an important hip extensor because of its large muscle volume, physiological cross-sectional area, and hip extension moment arm (Németh and Ohlsén, 1985; Ward et al., 2009), the relative contribution of this muscle may not have increased to the same extent as that of the semitendinosus as fatigue accumulated. A recent study by Takahashi et al. (2025) demonstrated that the torque-generating potential of the adductor magnus was more than twofold greater for hip extension than for adduction, and that its hip extensor torque-generating potential was correlated with maximal hip extension torque. It is well established that hip extension torque is a major contributor to forward acceleration during sprinting (Dorn et al., 2012; Morin et al., 2015; Higashihara et al., 2018; Nagahara and Murata, 2024). Moreover, because the hip extensor moment arm progressively increases with increasing hip flexion angle (Németh and Ohlsén, 1985), the larger hip extensor moment arm of the adductor magnus may play a primary role in generating hip extensor torque during the late swing and early stance phases of sprinting, when the hip is markedly flexed (Wiemann and Tidow, 1995; Nagahara and Murata, 2024). Consistent with these findings, the adductor magnus, like the semitendinosus, shows greater hypertrophy in sprinters than in untrained individuals relative to other thigh muscles (Handsfield et al., 2017; Miller et al., 2021; Takahashi et al., 2022). Therefore, the present findings suggest that adductor magnus activation during sprinting may play an important synergistic role in generating greater hip extension torque for superior sprint performance in sprinters.

Previous studies using T2-weighted MRI have reported non-uniform intramuscular activation in several thigh muscles following resistance exercise, running, and sport-specific movements (Akima et al., 2004; Mendez-Villanueva et al., 2016; Mechó et al., 2024). However, to the best of our knowledge, such intramuscular heterogeneity has not been investigated in response to maximal sprinting. The results of the present study showed significant regional differences in sprint-induced T2 changes in six thigh muscles (i.e., the rectus femoris, vastus lateralis, adductor magnus, semimembranosus, semitendinosus, and the long head of the biceps femoris), indicating non-uniform intramuscular activation that may reflect region-specific neuromuscular activity and metabolic loading within each muscle. Therefore, this study is also the first to determine the intramuscular regional distribution of sprint-induced thigh muscle activation. Previous studies using T2-weighted MRI have demonstrated regional differences in muscle activation following various exercise tasks in most of the thigh muscles that exhibited non-uniform intramuscular activation in the present study, with the exception of the adductor magnus (Akima et al., 2004; Mendez-Villanueva et al., 2016; Mechó et al., 2024). For example, Akima et al. (2004) reported greater activation in the distal region than in the proximal region of the rectus femoris after knee extension exercise. Mechó et al. (2024) reported greater activation in the middle and distal regions than in the proximal region of the semitendinosus after a soccer match. Furthermore, Mendez-Villanueva et al. (2016) reported that muscle activation in the long head of the biceps femoris increased progressively from the proximal to the middle and distal regions after hip extension exercise. In contrast, they also showed that, after knee flexion exercise, muscle activation in the long head of the biceps femoris increased progressively from the distal to the middle and proximal regions. Therefore, in light of their findings, non-uniform intramuscular regional distribution observed in the long head of the biceps femoris in the present study may have depended more on hip extension than on knee flexion during sprinting. Although the present study did not examine the relationship between sports injuries, particularly hamstring strain injuries, and thigh muscle activation patterns, these findings may help explain, at least in part, the mechanism underlying the high incidence of injuries to the proximal long head of the biceps femoris in sprinters (De Smet and Best, 2000; Askling et al., 2007). Taken together, the present findings not only demonstrate non-uniform intramuscular regional distributions of sprint-induced thigh muscle activation but also provide new insights into factors potentially contributing to hamstring strain injuries in sprinters.

## Limitations

This study has several limitations. First, we initiated post-exercise T2-weighted MRI acquisition approximately 5 min after the completion of the low-volume sprint protocol. Thus, the post-exercise T2 values measured in this study may not necessarily represent the peak responses immediately after exercise, and this time delay may have affected the magnitude of the observed T2 changes. Previous studies have suggested that post-exercise T2 recovery kinetics may be influenced by muscle fiber composition, as indicated by differences among rodent muscles and between trained and untrained individuals (Prior et al., 2001; Reid et al., 2001). However, muscle fiber composition has been reported to be relatively similar among thigh muscles (Garrett et al., 1984; Johnson et al., 1973). Furthermore, because all participants in the present study were sprinters, differences in muscle fiber composition attributable to training status were likely to be relatively small. In addition, differences in muscle fiber composition among human skeletal muscles may have a relatively limited influence on T2 recovery kinetics (Varghese et al., 2015). Therefore, although some recovery of T2 may have occurred during the approximately 5-min interval before post-exercise MRI acquisition, the influence of this time delay on the intermuscular and intramuscular comparisons of T2 changes in the present study may have been limited.

Second, we did not include the pelvic muscles, particularly the gluteus maximus, even though its activity and size are important factors for superior sprint performance in sprinters (Kyröläinen et al., 2005; Bartlett et al., 2014; Miller et al., 2021; Tottori et al., 2021). Indeed, Yoshimoto et al. (2022) evaluated sprint-induced muscle activation of the gluteus maximus using T2-weighted MRI. However, the primary aim of the present study was to examine intermuscular and intramuscular distributions of thigh muscle activation. To appropriately assess gluteus maximus activation, the muscle belly could be imaged at the level of the greater trochanter (Tottori et al., 2021); however, with the imaging range used in the present study, this level could not be included in most participants. In addition, if the gluteal muscles were assessed in the supine position, it may not be possible to obtain valid sprint-induced T2 changes, because muscle morphology could be altered by external compression, as indicated by the methodology of our morphological study and previous findings (Tottori et al., 2021; Shiotani et al., 2024). Therefore, further studies are needed to optimize the measurement sites and body position conditions to enable simultaneous assessment of the thigh muscles and gluteal muscles, particularly the gluteus maximus, thereby allowing comparisons of individual muscle activation as well as evaluations of intermuscular and intramuscular distributions across and within these muscles.

Third, because only male sprinters were included, the findings should be interpreted as specific to male sprinters. In fact, sprint kinematics and kinetics are widely known to differ between male and female sprinters (Slawinski et al., 2017; Nagahara, 2023). Furthermore, considering the potential relationship between acute muscle activity and chronic muscle hypertrophy (Wakahara et al., 2012, 2013, 2017), the muscles that specifically contribute to superior sprint performance in female sprinters may partly differ from those in male sprinters (Miller et al., 2021, 2022). Based on these findings, it is reasonable to speculate that T2-weighted MRI-measured muscle activation patterns may differ between male and female sprinters following maximal sprinting. Therefore, further studies are needed to include female sprinters and to investigate sex-related differences in muscle activation patterns during maximal sprinting.

Forth, although we discussed the associations of T2-weighted MRI-measured thigh muscle activation patterns with biomechanical and morphological variables in the results of the present study, we did not present these data. For example, because hip and knee joint kinematics and kinetics were not quantified during sprinting in the low-volume sprint protocol, we could not clearly indicate the impact of biomechanical factors on the specific patterns of thigh muscle activation observed in this study. In addition, although we assumed that greater size in specific thigh muscles is related to superior sprint performance, we did not examine the relationships between thigh muscle size and sprint performance in the participants in the present study. Therefore, we were unable to draw a clear conclusion regarding the present findings. To address this limitation, further studies are needed to adopt approaches that combine motion analysis and muscle morphology assessment with T2-weighted MRI-measured muscle activation in order to more comprehensively understand how biomechanical and morphological variables influence sprint-induced muscle activation patterns, as well as the reverse direction of these relationships. Such investigations may contribute to advancing our understanding of sports science and exercise physiology.

## Conclusion

This study examined the intermuscular and intramuscular distributions of sprint-induced thigh muscle activation, as assessed by the T2-weighted MRI, in response to maximal sprinting in male sprinters using an original low-volume sprint protocol. We demonstrated that this protocol was sufficient to detect pre- to post-exercise increases in T2 in the thigh muscles without impairing sprint performance across repeated maximal sprints, and that T2 increase-based muscle activation was markedly greater in the hamstrings and adductors than in the quadriceps femoris, particularly in the semitendinosus and adductor magnus, respectively, partly consistent with findings from pioneering studies (Bourne et al., 2021; Yoshimoto et al., 2022). Furthermore, this study was the first to show that sprint-induced thigh muscle activation was non-uniform not only across muscles but also across regions within specific muscles. In summary, the present findings suggest that maximal sprint performance is achieved through complex muscle recruitment mechanisms characterized by intermuscular and intramuscular distributions of thigh muscle activation, and that better understanding of these features may provide a basis for the development of novel sprint strategies in sprinters.

## Data Availability

The original contributions presented in the study are included in the article/supplementary material. Further inquiries can be directed to the corresponding author.
